# Design and validation of a simulated multitasking environment for assessing the cognitive load on the infantry squad leader

**DOI:** 10.3389/fpsyg.2024.1433822

**Published:** 2024-08-27

**Authors:** Alexis Remigereau, Françoise Darses, Baptiste Dozias, Julie Albentosa

**Affiliations:** Department of Neurosciences and Cognitive Sciences, French Armed Forces Biomedical Research Institute, Brétigny-sur-Orge, France

**Keywords:** Simulation, microworld, task analysis, convergent validity, content validity, military, dismounted infantryman

## Abstract

The increasing cognitive load on infantry squad leaders is a common challenge in modern military operations. As this can increase health and safety risks, there is a need to study the factors responsible for the increase in cognitive load. Ecological situations inherently lack strong experimental controls; therefore, microworlds that simulate real tasks are the usual alternative to field studies. However, to the best of our knowledge, there are currently no microworlds that reproduce the main tasks of the squad leader during operations. This article adresses this gap by describing the design and validation of a new microworld: the Simulated Multitasking Environment for the Squad leader (SMES). Qualitative research was firstly conducted to highlight several squad leader’s generic tasks (i.e., common to many situations in the field) that guided the design of the SMES. Psychometric validation of the SMES was then based on two experiments: (i) the first evaluated the microworld’s psychometric qualities when tasks were performed individually; and (ii) the second explored concurrent tasks, reflecting real-world complexity. The results showed that the parameters manipulated for each task were relevant for inducing cognitive load, measured using a secondary detection response task and the NASA-TLX questionnaire. The SMES demonstrated satisfactory convergent and content validity in multitasking but not in single-task conditions. Performance in multitasking situations therefore does not seem to depend on task-specific skills, suggesting the existence of an independent factor–multitasking ability. Theoretical and practical implications of the SMES validation are discussed.

## 1 Introduction

The equipment currently used by soldiers during operations is more sophisticated than ever. Not only do its powerful, multimodal functions make multiple cognitive demands, but dismounted soldiers (i.e., operates on foot rather than in a vehicule) also handle a multitude of tasks that must be carried out concurrently. This can lead to an increase of cognitive load, commonly defined as the result of a balance between the resources mobilized by an operator and the task demand (Moray, 1967). Cognitive load is a fuzzy, polysemous concept whose conceptualization and measurement encounter numerous obstacles (Young et al., 2015). Cognitive load is sometimes described as unidimensional (Tattersall and Foord, 1996) or multidimensional (Matthews et al., 2015b), and is subject to dissociations in its measurement (Hancock and Matthews, 2019). Cognitive load has recently been defined as is “the degree of activation, of a finite pool of resources, limited in capacity, while cognitively processing a primary task over time mediated by external dynamic environmental and situational factors, as well as affected by static definite internal characteristics of a human operator, for coping with static task demands, by devoted effort and attention” (Longo et al., 2022). This recent, comprehensive and inclusive definition highlights the mechanisms underlying the notion of cognitive load and is relevant to multitasking situations. It takes into account individual characteristics, in our case the squad leader and his specific characteristics, and the existence of several sources of demand mobilizing the same attentional resources (Oberauer, 2019). These include storage vs. processing (processing load), perception vs. memory (i.e., perceptual and memory load) and automatic vs. controlled attention (Oberauer, 2019). Numerous tools exist for measuring cognitive load in multitasking situations, including subjective, behavioral, physiological and performance measures (Cain, 2007). Among performance indicators, performance on a secondary task is popular approach (Cain, 2007). The detection response task, in particular, is known to be highly sensitive to variations in the level of cognitive load at various tasks (Stojmenova and Sodnik, 2018). It exists in several perceptual modalities, including visual, auditory and tactile (Stojmenova and Sodnik, 2018). To assess the effects of perceptual modality on cognitive load in multitasking situations, Hollands et al. (2019) asked soldiers to perform tasks whose demands varied according to the speed of message presentation (fast; slow) and their perceptual modalities (auditory; visual). The results showed that reaction times were shorter when message presentation was slow and when messages were presented audibly. In a real-life multitasking situation, this modality effect on cognitive load could be influenced by other cognitive processes involved in performing other operational tasks. An experiment taking these other cognitive processes into account would provide a higher level of external validity and a better assessment of the benefits of using multimodal navigation and communication equipment for squad leaders.

The squad leader must supervise a team of 6 to 7 soldiers, and is regularly involved in multitasking situations. Examples include coordinating teams during a tactical action while maintaining a permanent link with their superior, or receiving orders by radio while gathering geolocation information and remaining aware of changes in their environment. Due to concurrent multitasking, squad leaders are particularly vulnerable to cognitive overload, which appears when the individual’s resources are not sufficient to cope with task demands, leading to performance degradation (Schneider and Shiffrin, 1977). Thus, cognitive load is likely to be a risk factor. This risk has been stressed by many authors (see Chérif et al., 2018), and it is a major concern for military staff. Multitasking situations exist in three forms (Salvucci and Taatgen, 2011), and infantry group leaders are confronted with all three in their work. The first form, concurrent multitasking, involves performing tasks simultaneously that compete for attentional (Kahneman, 1973) and working memory resources (Baddeley, 2003). The second, task switching, involves performing one task at a time, switching from one to another by alternating task sets (Monsell, 2003). Models have been proposed to describe attentional resource allocation strategies in task-switching situations, such as the STOM model (Wickens et al., 2015). The third form of multitasking is task interruption (Altmann and Trafton, 2002). The memory for goal model provides a framework explaining how a task in progress is interrupted, stored in working memory and then resumed (Altmann and Trafton, 2002). Concurrent multitasking, in particular, is the form of multitasking that generates the greatest cognitive load (Wickens, 2002, 2008). Debates exist as to whether information processing capacities are resource-based (i.e., energetic-based model; Kahneman, 1973) or structural, based on a bottleneck (Broadbent, 1958). The difference lies in whether it is really possible to process more than one piece of information at a time (Navon and Miller, 2002), with some evidence showing that it is (Maquestiaux et al., 2008) and some showing that it is not (Rau and Zheng, 2020). For energetic-based models in particular, paradigms clash between those defending the existence of a single pool of attentional resources (Kahneman, 1973), while others defend the idea of multiple attentional resources (Wickens, 2002, 2008). Providing answers to these debates is crucial to the study of the cognitive load of military personnel and to provide effective recommendations for, for example, the design of new communication and orientation equipment inducing a lower cognitive load.

Performance degradation due to a high cognitive load is difficult to measure using field data. The environmental constraints (dynamic situation, irregular relief, variation in light, etc.) prevent the deployment of instruments that could capture the fine-grained data required for cognitive analysis (eye tracking, physiological measurements, etc.). It calls for the design of microworlds that enable the cognitive conditions of field activity to be reproduced in simulated task environments. The work we present here extends this line of research. We designed and validated a simulated multitasking environment, called SMES (Simulated MultiTasking Environment for the Squad Leader). This microworld will make it possible to measure the cognitive cost of the multitasking situations faced by dismounted soldiers, more specifically, the squad leader. Our aim was to develop a simulated multitasking environment that is representative of the real task usually encountered by the squad leader during operations. It will thus be possible to vary the characteristics of the simulated tasks (e.g., their level of difficulty) to study the impact on the tasks performance. Beginning in the 1990s, research on the design of simulation environments has been particularly fruitful, under the heading of simulated task environments or microworld (Brehmer and Allard, 1991; Brehmer and Dörner, 1993; Gray, 2002). Cañas and Waern (2005) define a microworld as “an environment created for making systematic research studies within a complex domain (…) where only aspects of the environment that are considered to be important from a research point of view are selected. (…) In these environments, the experimenter is able to design situations that reflect realistic conditions of decision-making and problem-solving without losing experimental control.” Simulated task environments are designed to study phenomena occurring in the real world, and researchers who build these are primarily interested in generalizing their findings back to the original task (Gray, 2002). All simulated task environments have different levels of correspondence, i.e., the degree of analogy of the simulation with its real-world equivalent (Gray, 2002). A low level of correspondence means that the simulated task environment reproduces few characteristics common to many task environments, while a high level means that it reproduces many characteristics of few or even one environment (Gray, 2002). In the study of simulated task environments, a key distinction lies in the types of tasks employed: generic tasks and specific tasks. Generic tasks are tasks that are broadly applicable across various scenarios and may not be directly tied to a particular situation, allowing for more generalizable findings (Cooke and Shope, 2004; Gray, 2002). On the other hand, specific tasks are designed to closely mimic real-world tasks in particular situation, exercising skills, cognitive processes and expertise relevant to those specific contexts within a controlled setting (Cooke et al., 1999). While specific tasks offer a more targeted investigation of particular real-world tasks, generic tasks provide a more relevant approach for studying fundamental cognitive processes (Cooke and Shope, 2004). Both, however, can bridge the gap between controlled laboratory studies and uncontrolled field studies (Cooke and Shope, 2004). In the case of this study, a simulated task environment based on generic tasks is therefore more appropriate, the aim being to achieve a high level of external validity and draw conclusions that can be generalized to various operational situations. Today, microworlds are designed either for training purposes or for research on cognitive processes. Using simulated environments, dismounted soldiers can be trained in basic tasks such as locomotion on open terrain, changing view and orientation while in position, weapons’ calibration and use, and visual recognition (Knerr, 2006). Microworlds can also be used to train military personnel in more complex missions. Examples include assault tasks (Silk and Billing, 2013), military operations in urban terrain (MOUT), securing a food convoy route, or leading a search for arms and contraband (Kaber et al., 2013). Most microworlds have been developed context free like the SYNWORK environment (Elsmore, 1994) or context specific, like the Multi-Attribute Task Battery-II (MATB-II; Cegarra et al., 2020; Comstock and Arnegard, 1992; Santiago-Espada et al., 2011). It simulates five piloting tasks (e.g., maintaining course, checking fuel consumption, or managing radio communications). The experimental conditions of these tasks can be varied in terms of simultaneity, allowed response time, presentation duration, or difficulty of execution. The properties of these tasks are intended to reproduce the processes used by a pilot: tracking, system monitoring, resource management, etc. However, these simulated tasks are not similar to those performed by squad leaders. Moreover, they never seem to have been empirically validated, as no study has shown that observed performance with the MATB-II is predictive of performance on the pilot tasks it is supposed to simulate. Its design approach has also never been described. Thus, MATB-II is not transposable to the cognitive constraints of the squad leader. As far as we know, there is no microworld specifically designed to carry out research with squad leader. To study cognitive processes of squad leaders in multitasking situations, the design of such a tool is necessary.

Another issue related to microworlds is to improve the psychometric and validity of simulated environments. As Harris et al. (2020) point out, “it is necessary to first establish whether the simulation captures fundamental features of the real task and environment.” The concept of ecological validity is rarely defined, and when it is, there is no consensus (Holleman et al., 2020). The concept of external validity was favored and refers to the possibility that the results of a study or a conclusion, for example obtained following an experiment, can be generalized to other tasks, or even other situations (Campbell, 1957; Holleman et al., 2020). In our case, operational infantry situations. To ensure the external validity of the results observed with SMES, several criteria must be met, such as the study population (i.e., squad leaders), task selection (e.g., determined using a qualitative approach) and multitasking (i.e., tasks competing in an operational situation). The work presented in this article pursues this goal. Our aim is to identify the cognitive mechanisms that need to be taken into consideration in multitasking situations. Further, recommendations will be drawn up for future equipment to ensure that operational safety is not compromised. We thus designed the SMES microworld to be a simulated multitasking environment for the dismounted squad leader. We adopt a comprehensive approach. First, we performed an analysis of the real-life activity of squad leaders. Second, we designed the experimental tasks. Third, we proceed to the psychometric validation of the SMES by conducting two complementary experiments.

## 2 Design of a simulated multitasking environment for the squad leader

The SMES design process was divided into three stages: (i) an analysis of the real-life activity of dismounted squad leaders in situations with high cognitive load; (ii) the development of generic tasks; and (iii) mapping these generic tasks to laboratory tasks, which have been validated.

### 2.1 Step 1: analysis of the real-life activity of dismounted squad leaders in situations with high cognitive load

We adopted a qualitative approach, focusing on the analysis of field activity through 27 semi-structured interviews and filming 32 h of operational activity during training. The aim was to identify situations that generally generate a high cognitive load. In a first phase, we analyzed the content of the interviews, which enabled us to draw up a list of 17 typical situations (e.g., being in contact with the enemy, losing the radio link, caring for the wounded). For reasons of confidentiality, the complete list of these situations is not provided here. In the second phase, we examined the videos and extracted sequences relating to these typical situations. This process resulted in a 12-h video corpus, from which we extracted and analyzed behaviors and communications using a coding scheme composed of generic tasks. The analysis was conducted using the Observer XT (Noldus) software package. Each time a generic task from the coding scheme was observed in a squad leader, it was coded with start and end times, allowing for overlapping categories. The coding scheme was developed from standard military procedures that describe the basic actions that infantry personnel must perform (Ministère de la Défense, 2008). Additionally, it incorporated elements from three other taxonomies developed from task analysis: one categorize communication types relevant to situational awareness (e.g., giving an order, reporting, etc.) (Christ and Evans, 2002), another identifying core infantry activities for training tool development (e.g., navigation, communication, surveillance, etc.) (Curtis and Hobbs, 1997) and a third focusing on core activities to enhance information presentation (e.g., observing, communicating, controlling fire, etc.) (Tack and Angel, 2005). Redundant categories were identified and removed. To ensure reliability, inter-rater agreement was calculated on 25% of the collected data using Cohen’s Kappa (Cohen, 1960, 1968). It confirmed that coding was reliable (all coefficients >0.61). Analyzing the training video sequences using the coding scheme enabled us to check whether it was robust (i.e., inter-rater agreement) and exhaustive, that is any new categories needed to be added, and this was not the case. The complete list of generic tasks is shown in Table 1.

**Table 1:** Generic tasks of the coding scheme.

Generic tasks
Receiving information
Receiving an order
Reporting
Analyzing teams’ tactical situation
Giving an order
Informing its teams
Receiving a report
Interacting with the threat
Shooting
Aiming
Walking forward
Protecting oneself
Analyzing the tactical situation of another squad
Giving tactical information to another squad
Spatial orientation

### 2.2 Step 2: identification of generic task groups

The generic tasks share several common characteristics. For example, the generic tasks, “*Receiving an order*” and “*Giving an order*” all relate to one and the same group that we named “*Communication with subordinates and superiors*.” This process made it possible to create four generic task groups, which are present in all typical situations that generate a high cognitive load. As indicated by (Harris et al., 2020), a simulation tool aims to capture “key features” of real task, rather than imitate it exactly. In our study, we postulate that the generic tasks contained in the four groups constitute the key features of the real tasks that will be simulated. If the generic tasks and laboratory tasks implemented in SMES share the same key features, the simulation tool should be able to rely on cognitive processes identical to those mobilized by dismounted squad leaders in a real-life situation. These laboratory tasks may draw upon cognitive functions that are identical to those mobilized by dismounted squad leaders in a real-life situation:

As the coding scheme has been shown to be robust and exhaustive, we have divided the generic tasks into four groups:

- Group 1: “Monitor and control the environment to detect any change in the tactical situation,” comprising the generic tasks “receiving information,” “informing its teams,” “analyzing the tactical situation of another squad” and “giving tactical information to another squad.” This group outlines the characteristics of a task for monitoring changes in the tactical situation and informing the individuals concerned of these changes.
- Group 2: “Spatial orientation using standard tools (compass, map, etc.)” is based on a single generic “spatial orientation” task, but involves several features. It involves using specific equipment such as a compass and a map or GPS to estimate a direction, distance or a location in the environment.
- Group 3: “Communication with subordinates and superiors” comprises the generic tasks “receiving an order,” “reporting” and “receiving a report.” This generic task group suggests an information exchange task between the squad leader and his subordinates, as well as with his superior. This involves the analysis and memorization of information which must be transmitted to a higher or lower echelon via the radio network.
- Group 4: “Tactical decision-making to position teams (on patrol, in contact, etc.)” includes all remaining generic tasks such as “analyzing teams’ tactical situation,” “giving an order,” “shooting,” “aiming,” “walking forward” and “protecting oneself.” This group outlines the characteristics of a tactical decision-making task based on an analysis of visuo-spatial features (i.e., position of friends, enemies, route, etc.).

### 2.3 Step 3: mapping the four generic task groups to laboratory tasks

In a real-life situation, the tasks identified above are often interdependent. For example, spatial orientation may require the squad leader to contact subordinates in order to obtain geolocation data. This interdependence makes it impossible to measure performance specific to each of the two tasks. To avoid this pitfall, we decided to match each generic task group with a laboratory task reflecting similar task features. In selecting tasks, priority was given to those designed for the infantryman, and if so, validated. Validity here means that the study has demonstrated the task’s ability to predict a relevant criterion of operational situations.

**Figure 1:**
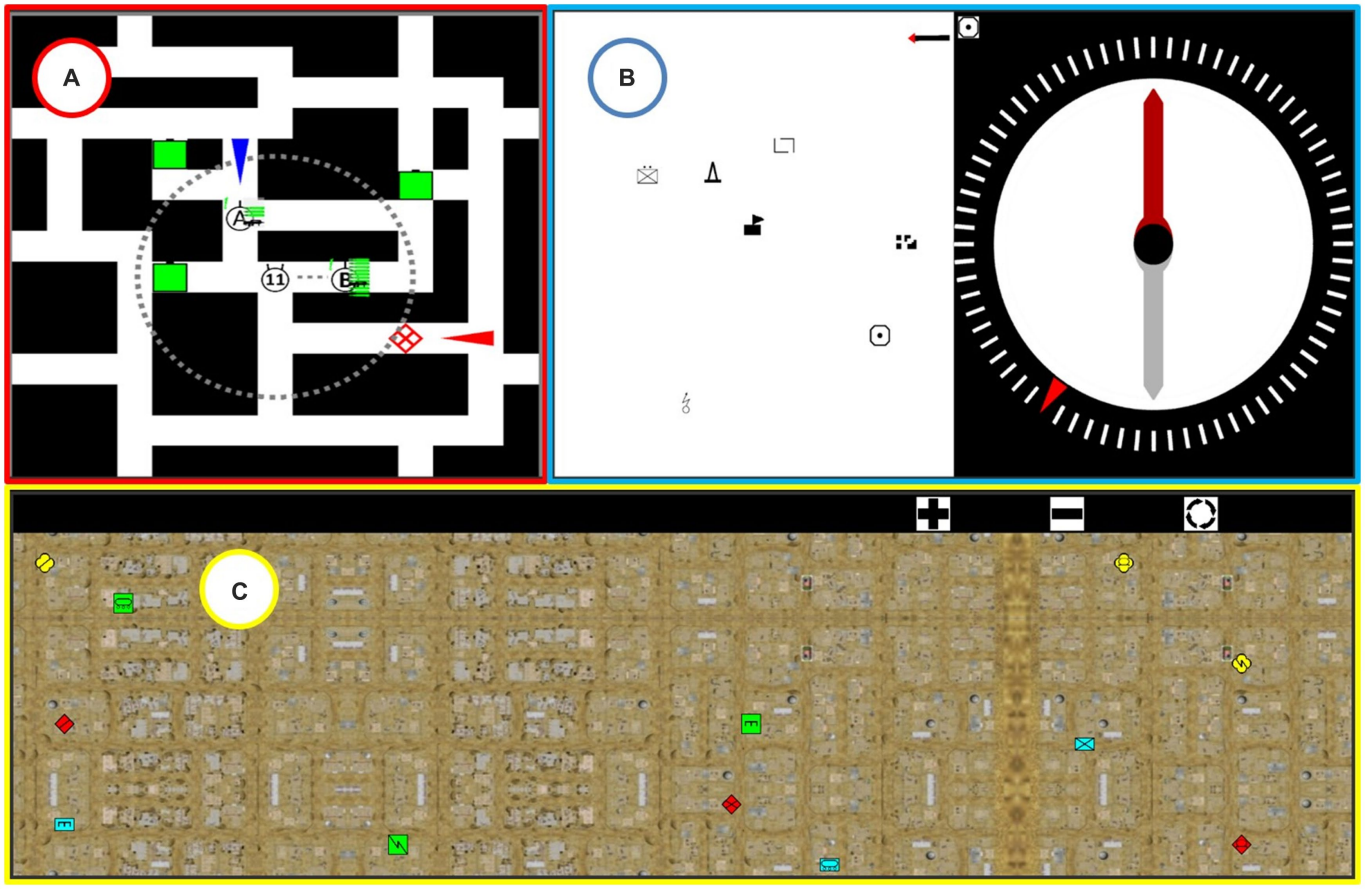
Screenshot of the simulated task environment. **(A)** The tactical decision-making task is shown at the top-left and framed in red. **(B)** The modified perspective-taking and spatial orientation task is shown at the top-right and framed in blue. **(C)** The change detection task is framed in yellow and displayed at the bottom of the screen. As the military-specific auditory N-back task (MSANT) is an auditory task, it is not shown here.

For Task 1 (Monitor the environment) and Task 3 (Communication), we were able to identify from the literature some laboratory tasks that accurately matched their task features. For Task 2 (Spatial orientation), we were able to identify a task that partially matched the task features; we therefore took it as a basis, and modified it. For Task 4 (Tactical decision-making), we were unable to identify a corresponding task in the literature. We therefore designed a new task based on the analysis of the activity. We provide details of the content of these four tasks below. Their representation in the SMES is shown in Figure 1.

- Task 1 (T1) Change detection: Designed by Matthews et al. (2015a), this task presents the participant with military symbols (e.g., a green square) distributed on a topographical map. Three types of change can occur randomly: appearance, disappearance, or change in the location of one or more symbols. Each time a change is detected, the participant must press a button (a button box). The performance indicator is the rate of correct responses: the sum of correctly detected appearances, disappearances, and changes in location. This task was chosen because symbol events represent changes in the tactical situation, while the response to the detection of an event can represent the information given to its teams.
- Task 2 (T2) Modified Perspective-Taking and Spatial Orientation (PTSOT): This task is inspired by the perspective-taking and spatial orientation test (Hegarty, 2004). The participant must indicate the cardinal direction of one map symbol in relation to another, with reference to the north, indicated by an arrow. He must respond by manipulating the dial of a virtual compass. The performance variable is the mean angular error (MAE), corresponding to the mean of the difference between the correct direction and the direction indicated by the participant in each trial (with a maximum error of 180°). This task was chosen because perspective-taking is very common for infantrymen in operations, and PTSOT performance correlates with navigation performance in operational situations (Johansson et al., 2014).
- Task 3 (T3) Military-Specific Auditory N-back Task (MSANT): This task was designed by Vine et al. (2022). The participant is presented with several sets of phonetic NATO letter pairs (alpha-bravo, delta-golf, etc.). The pairs are spoken. Immediately after hearing an auditory signal, the participant is asked to orally recall one of the pair of letters they have heard (the last pair, the penultimate pair, etc.). The performance variable is the percentage of letter pairs correctly recalled. The MSANT task was chosen because it closely mimics aspects of military radio traffic, such as the use of the NATO phonetic alphabet (Vine et al., 2022).
- Task 4 (T4) Tactical decision-making: This task was newly designed, due to the inability to identify a task specific to military and visuo-spatial tactical decision-making in the literature. The participant is presented with a maze that contains two teams of infantry soldiers (A and B), their enemies (one or two per trial), and targets (presented as green squares). The participant is asked to order the infantry teams to neutralize the enemies before they reach the targets. The performance variable is the success rate (the mean rate that enemies are neutralized, and targets are successfully protected).

## 3 Experimental validation of the SMES

The psychometric validation process was guided by three key references. Ehret et al. (1998) introduced a model with three dimensions crucial for the effectiveness of simulated task environments. The first dimension, tractability, refers to how well the research question aligns with the characteristics of the simulated task environment. In our study, this means the tool must induce varying levels of task demand to examine its effect on cognitive load. The second dimension, realism, relates to the environment’s ability to replicate the key features of real tasks. The third, engagement, concerns the participant’s motivation to interact with the simulation tool. Harris et al. (2020) provided a framework for psychometrically validating simulated task environments that replicate certain real-world task features. This framework particularly assesses convergent and content validity. Additionally, Matthews et al. (2015a) proposed a taxonomy of criteria for validating cognitive load measurement tools, focusing on sensitivity and reliability. These criteria enable the assessment of a tool’s ability (e.g., performance on detection response tasks, NASA-TLX scores) to detect cognitive load variations, and the relevance of the manipulated parameters for each task. Specifically, the criteria outlined by Matthews et al. (2015a) help evaluate the tractability of SMES, while Harris et al.’s (2020) framework assesses realism through convergent validity and engagement through content validity. To validate the SMES, we conducted two complementary experiments. The first assessed each primary task individually, while the second verified the SMES’s validity in multitasking situations.

Three psychometric criteria were verified:

- Sensitivity of measures: This refers to the ability of measurements to detect variation in cognitive load as a function of a given factor; in our case, the level of task demand;
- Content validity: This refers to verifying the representativeness of the tasks as perceived by experts, as well as their effectiveness in reproducing the main characteristics of real-life tasks.
- Convergent validity: This refers to ensuring that variables intended to measure the same construct are highly correlated with each other. In our case, marks obtained in professional examinations should be correlated with performance on the SMES tasks.

### 3.1 Experiment 1: validity of each task and sensitivity of the measures

#### 3.1.1 Objective

This first experiment assessed the validity of each SMES task on three dimensions: (i) the sensitivity of the measures to the cognitive load induced by the task; (ii) convergent validity; and (iii) content validity.

The theoretical hypotheses are that:

- An increase in cognitive load should be observed as task demands increase, reflecting a depletion of cognitive resources (Kahneman, 1973). This would indicate sufficient sensitivity of cognitive load measures (Matthews et al., 2015a; O’Donnell and Eggemeier, 1986);
- The SMES should have a good convergent validity if the performance on the SMES tasks are correlated to the marks obtained in professional examinations (Harris et al., 2020);
- The SMES should have a good content validity if the experts, i.e., squad leaders, perceive that the SMES tasks are representative of the tasks that they perform in real-life (Harris et al., 2020).

#### 3.1.2 Method

##### 3.1.2.1 Participants

Seventeen infantry squad leaders (14 men, 3 women) took part in the study. The mean age was 28.6 years (SD = 4.94); mean seniority in the military was 5.96 years (SD = 4.04), and squad leader seniority was 2.21 years (SD = 1.45). Selection criteria included being at least 18 years old, infantry squad leader status, at least one field experience as a squad leader (domestic or overseas operations) and normal vision and hearing. The studies involving human participants were reviewed and approved by the Research Ethics Committee POLETHIS affiliated with the Université Paris-Saclay (reference: CER-Paris-Saclay-2022-013). The studies were conducted in accordance with the local legislation and institutional requirements. The participants provided their written informed consent to participate in this study.

##### 3.1.2.2 Materials

###### 3.1.2.2.1 Simulated multitasking environment for the squad leader task characteristics as a function of demand

The four SMES tasks were manipulated to generate three demand levels. The characteristics manipulated for each task are shown in Table 2.

**Table 2:** Demand parameters manipulated for each task.

Task	Low demand	Medium demand	High demand
T1	20 stimuli to detect	40 stimuli to detect	80 stimuli to detect
T2	11 s. to answer	9 s. to answer	7 s. to answer
T3	1-back	2-back	3-back
T4	1 target to protect and 2 enemies in 25% of trials	2 targets to protect and 2 enemies in 50% of trials	3 targets to protect and 2 enemies in 75% of trials

###### 3.1.2.2.2 Detection response task (DRT)

The DRT (Stojmenova and Sodnik, 2018) is a secondary task that is often used to evaluate cognitive load. It is performed in parallel with another task whose cost is being assessed, and it involves responding as quickly as possible when a signal occurs, by pressing a button attached to the index finger. The signal can be visual, auditory or tactile. In this study, only a tactile signal was used, consisting of a vibration emitted by a chip attached to the forearm. Two indicators were recorded: the proportion of stimuli detected, and the reaction time. Only responses between 100 ms and 2,500 ms were analyzed; values beyond this range were considered to be false alarms. The tactile version of the DRT was chosen to minimize interference with visual and auditory tasks, and to ensure that all tasks were considered equally (Wickens et al., 1983; Wickens, 2002, 2008).

###### 3.1.2.2.3 The NASA-raw task load index (NASA-RTLX)

The NASA-raw task load index (NASA-RTLX; Georgsson, 2019) is a 6-item questionnaire that measures perceived cognitive load (mental demand, physical demand, time pressure, effort, frustration, and perceived performance), rated by respondents on a scale from 0 to 100. The mean overall score is then calculated.

###### 3.1.2.2.4 Content validity questionnaire

This *ad hoc* questionnaire is composed of 14 items (*cf.* Supplementary material) grouped into the four dimensions described below. Participants were asked to evaluate the system’s ability to reproduce the main characteristics of operational tasks carried out by infantry personnel:

- The dimension “Mental representation of the situation” (items Q1 and Q2): the perceived ability of the microworld to reproduce the difficulties and cues used in real-life situations to make tactical decisions.
- The dimension “Attentional costs of tasks” (items Q3 to Q6): the perceived similarity between the cost in terms of attentional resources generated by the microworld’s tasks and their real-life equivalents.
- The dimension “Cognitive processes mobilized” (items Q7 to Q11): the perceived similarity between the cognitive processes mobilized by the microworld’s tasks and those mobilized in real-life tasks (e.g., visuo-spatial processing, remembering information).
- The dimension “Usefulness and practicality” (items Q12 to Q14): the perceived relevance of using the microworld for training and evaluating the skills of infantry squad leaders.

Inter-rater reliability in questionnaire responses was assessed using an intra-class correlation coefficient. In cases where the correlation was poor, we decided to note the rate of positive responses (> 25/50) which better-represents the participant’s degree of agreement with each item.

The questionnaire was implemented using macros in Visual Basic for Applications (VBA; Microsoft) and filled in via a computer.

###### 3.1.2.2.5 Evaluating convergent validity

The following criteria were adopted to assess convergent validity: marks obtained by squad leaders in the Military Certificate Level 1 (MC1), and Technical Certificate Level 1 (TC1) exams. These exams include exercises that draw upon the same cognitive processes found in the SMES tasks. The course includes a number of technical and tactical skill modules that are linked to the conduct of operational missions, such as the ability to use topographic (map-terrain liaison, bearings, coordinates, radio transmission) or infantry-related knowledge.

##### 3.1.2.3 Experimental design and procedure

We adopted a repeated measures, 2 × 2 factorial design. Independent variables were Task Type (T1; T2; T3; T4) and Task Demand (low; medium; high). Dependent variables (DVs) were: performance (success rate), the detection rate of stimuli during the DRT, and the mean detection response time (RT). For the NASA-RTLX, the DV was the overall score. For the content validity questionnaire, DVs were scores indicated for each item. For convergent validity, the DV was the mark obtained by each participant in the two military examinations. Participants began by completing a questionnaire that collected biographical data. Written instructions for each task were then provided, and participants were invited to ask any questions they might have. For each task, they completed a familiarization phase. Participants were told to consider the DRT as a secondary task, and prioritize the microworld’s tasks. Participants then carried out the 12 experimental tasks in random order. Each task lasted 5 min. The NASA-RTLX was completed after each session. When all 12 tasks had been completed, the content validity questionnaire was filled in by participants.

##### 3.1.2.4 Statistical analysis

To assess the sensitivity of the measures, several repeated-measures ANOVAs were run to evaluate the effect of each task’s demand level on performance, DRT RTs, and NASA-RTLX scores. Mauchly sphericity and Shapiro–Wilk normality tests were run before each ANOVA. A Greenhouse–Geisser correction was applied when sphericity was not sufficient. A Friedman test and Conover *post-hoc* tests were run when the data did not follow the normal distribution. The Friedman test is in fact a recommended alternative to the repeated-measures ANOVA for non-normal data (Friedman, 1937; Xu et al., 2017).

Two tests were run to check content and convergent validity:

- Correlation tests (Bravais–Pearson) were used to evaluate convergent validity between task performance and exam marks.
- An intra-class correlation test was used to estimate inter-rater reliability in responses to the content validity questionnaire. This made it possible to determine whether means were sufficiently reliable to be interpreted, or whether it was preferable to evaluate the percentage of participants who indicated a value ≥25 for each item (Classen et al., 2020; Polit and Beck, 2006).

For all these analyses, the significance threshold was set at 0.05.

##### 3.1.2.5 Operational hypotheses regarding task-induced measurement sensitivity

To evaluate the ability of the measures to detect variation in cognitive load among participants, we manipulated task demand. The hypotheses are (H1a) as task demand increases (i.e., from low to high level), task performance should deteriorate, (H1b), mean DRT-RT (response time) should increase and (H1c) overall NASA-RTLX scores should increase.

##### 3.1.2.6 Operational hypotheses regarding convergent validity

Hypothesis H2 is as follows: There should be a correlation between performance obtained with the multitasking system, and MC1 and TC1 marks.

##### 3.1.2.7 Operational hypotheses regarding content validity

Hypothesis H3 is as follows: The simulator’s content validity will be considered adequate if, for each item on the questionnaire, scores are above 25/50 for at least 50% of participants.

#### 3.1.3 Measurement sensitivity results

##### 3.1.3.1 Effect of task demand on performance

**Figure 2:**
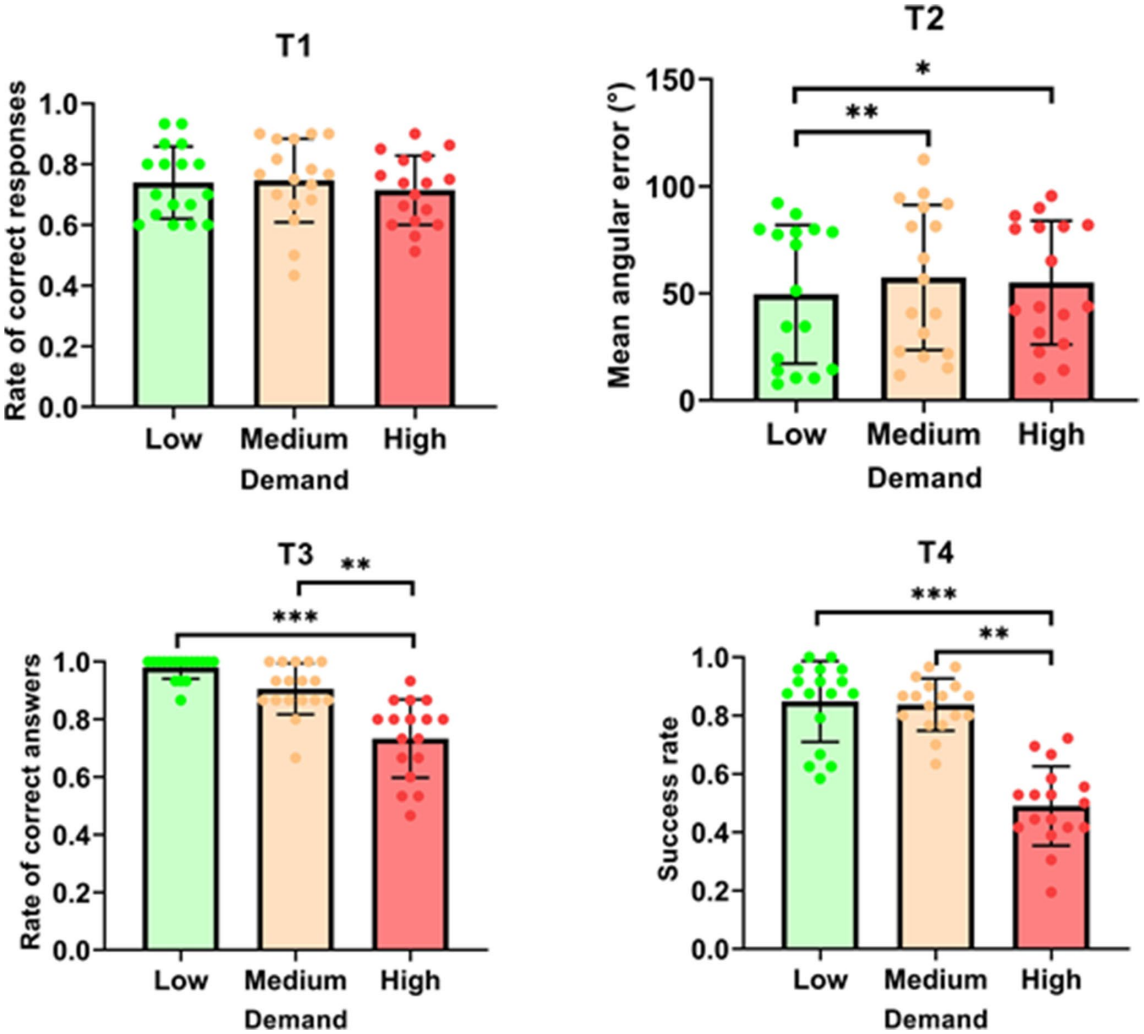
Mean performance for each task as a function of demand. *p < 0.05; **p < 0.01; ***p < 0.001.

The analysis for T1 revealed no significant effect of demand on the rate of correct responses [*F* (2, 32) = 0.672; *p* = 0.518; see Figure 2]. In contrast, T2 showed a significant effect of demand on mean angular error (MAE) [*χ*^2^ (2) = 10.941; *p* = 0.004]. The MAE was significantly higher in the ‘medium’ condition (57.39 ± 33.88) compared to the ‘low’ condition (49.58 ± 32.36, *p* = 0.004). Similarly, the MAE was also higher in the ‘high’ condition (55.02 ± 28.92) than in the ‘low’ condition (49.58 ± 32.36, *p* = 0.015). For T3, demand had a significant impact on the rate of correct answers [*χ*^2^ (2) = 24.121, *p* < 0.001]. Specifically, the rate of correct answers was higher in the ‘low’ condition (0.980 ± 0.039) compared to the ‘high’ condition (0.733 ± 0.135, *p* < 0.001). Additionally, the rate was higher in the ‘medium’ condition (0.906 ± 0.088) than in the ‘high’ condition (0.733 ± 0.135, *p* = 0.006). Similarly, T4 results indicated a significant effect of demand on the success rate [*χ*^2^ (2) = 24.121; *p* < 0.001]. The success rate was greater in the ‘low’ condition (0.848 ± 0.138) than in the ‘high’ condition (0.490 ± 0.136, *p* < 0.001). Moreover, the success rate in the ‘medium’ condition (0.837 ± 0.090) was also higher than in the ‘high’ condition (0.490 ± 0.136, *p* < 0.001). Overall, the results indicate that increased demand adversely affects performance in Tasks 2, 3, and 4, with higher error rates and lower success rates observed under medium and high demand conditions. However, T1 performance remained unaffected by changes in demand.

##### 3.1.3.2 Effect of task demand on the percentage of tactile stimuli detected during the DRT

**Figure 3:**
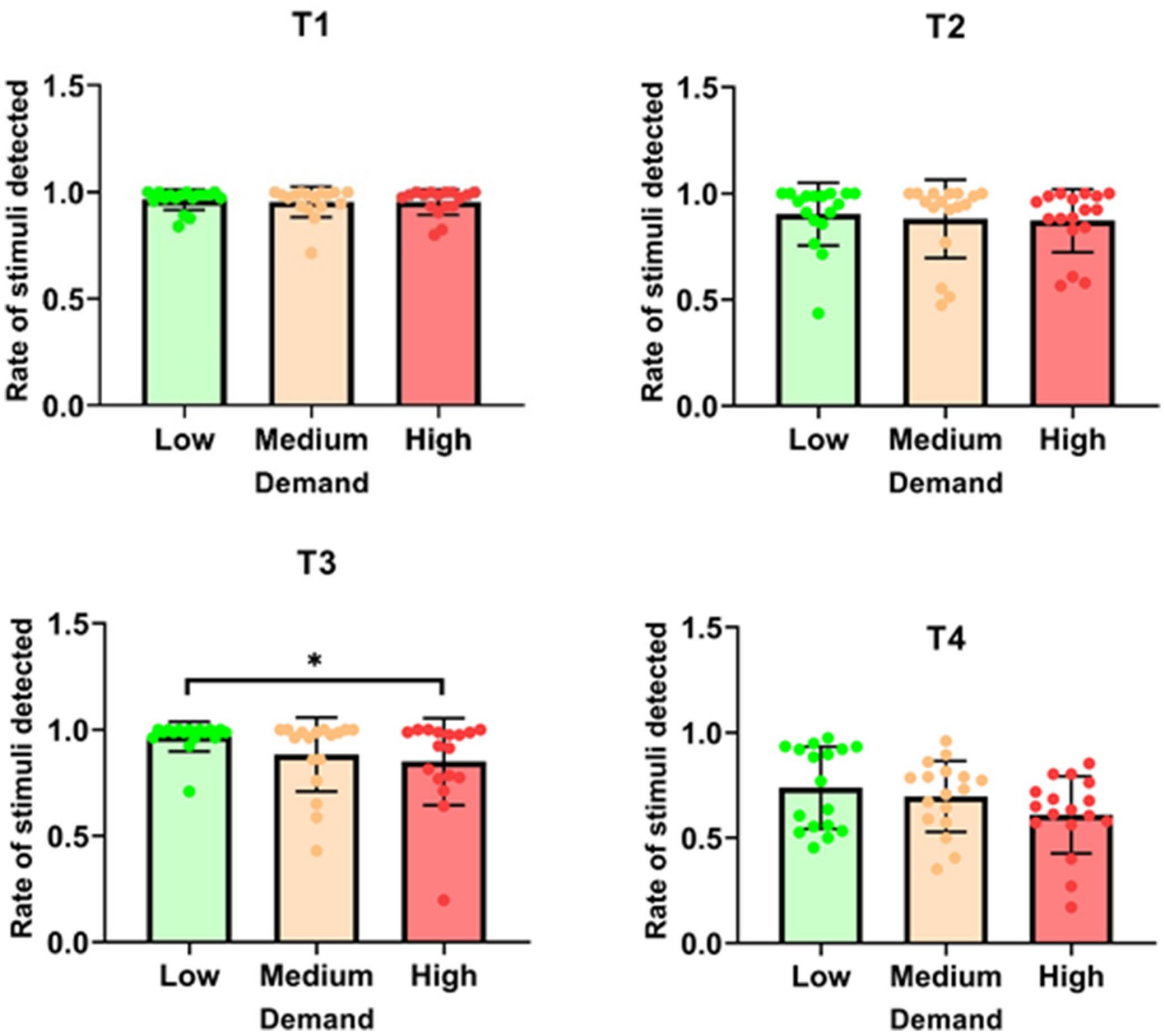
Percentage of tactile stimuli detected for each task as a function of demand. *p < 0.05; **p < 0.01; ***p < 0.001.

For T1, there was no significant effect of task demand on the rate of stimuli detected [*χ*^2^ (2) = 0.034; *p* = 0.983]. Similarly, T2 showed no significant effect of task demand on the detection rate [*χ*^2^ (2) = 3.263; *p* = 0.196] (see Figure 3). In contrast, T3 revealed a significant effect of task demand, as indicated by the Friedman test [*χ*^2^ (2) = 6.125; *p* = 0.047]. The rate of detected stimuli was significantly higher in the ‘low’ condition (*M* = 0.968 ± 0.070) compared to the ‘high’ condition (*M* = 0.850 ± 0.205, *p* = 0.028). For T4, the effect of task demand approached significance but did not reach the threshold for statistical significance [*χ*^2^ (2) = 5.765; *p* = 0.056]. Overall, the results indicate that task demand did not significantly affect the rate of stimuli detected in Tasks 1 and 2. However, for T3, higher task demand led to a reduced detection rate, and for T4, there was a marginal effect that suggests a potential trend toward reduced detection with increased demand.

##### 3.1.3.3 Effect of task demand on mean RT (ms) in the DRT

**Figure 4:**
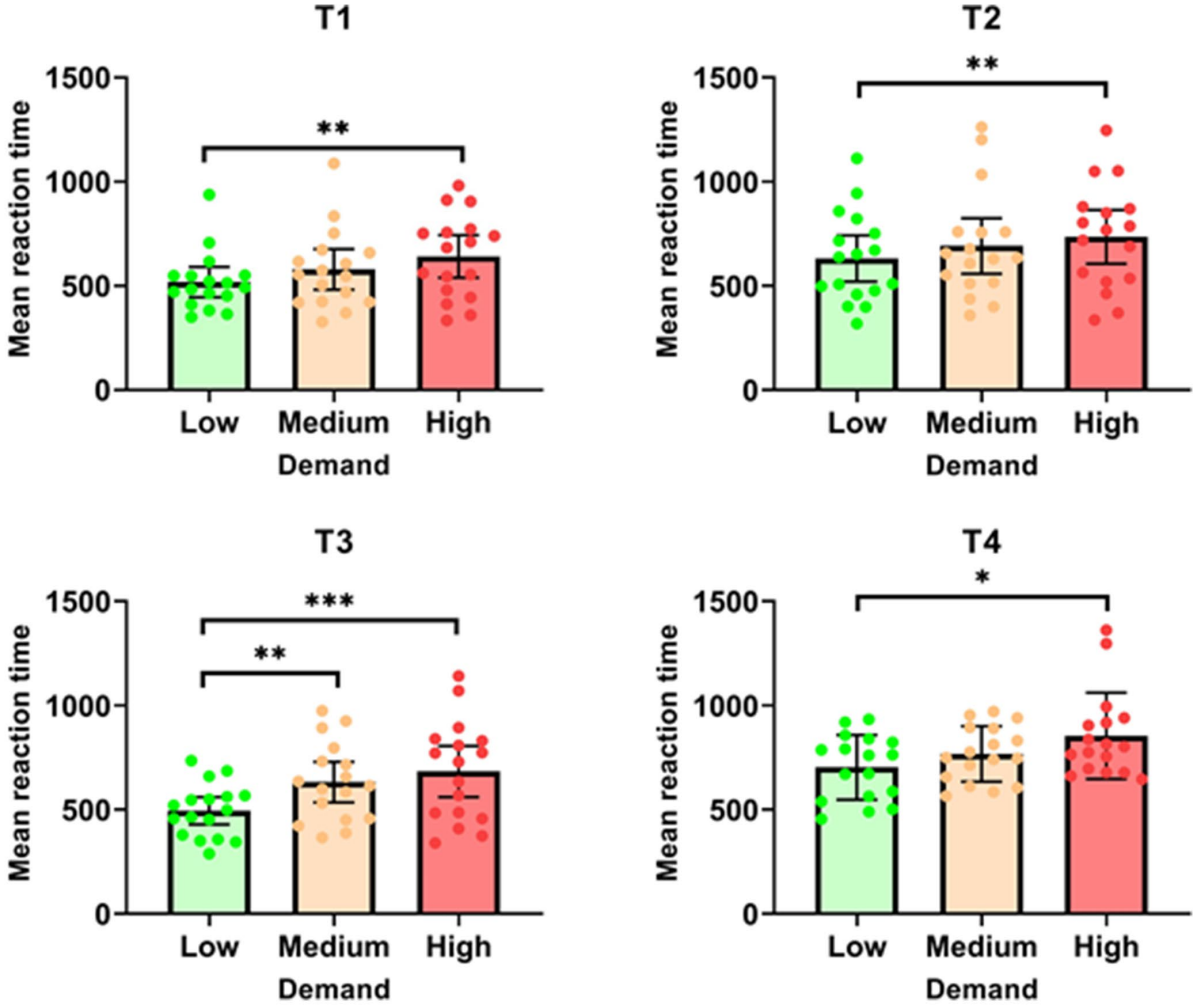
Mean RT for each task as a function of demand. *p < 0.05; **p < 0.01; ***p < 0.001.

The analysis of mean reaction times (RT) across different task demands revealed significant effects for all tasks (see Figure 4). For T1, there was a significant effect of task demand on mean RT [*F* (2, 32) = 6.157; *p* = 0.005]. Specifically, mean RTs were significantly higher in the ‘high’ demand condition (*M* = 641.17 ± 198.59) compared to the ‘low’ demand condition (*M* = 518.09 ± 141.06, *p* = 0.004). Similarly, for T2, a significant effect of demand on mean RT was observed [*F* (2, 32) = 7.448; *p* = 0.002]. Mean RTs were significantly higher in the ‘high’ condition (*M* = 735.13 ± 250.59) than in the ‘low’ condition (*M* = 630.91 ± 215.95, *p* = 0.002). T3 also showed a significant effect of task demand on mean RT [*F* (2, 32) = 13.854; *p* < 0.001]. Mean RTs were significantly higher in the ‘medium’ condition (*M* = 631.51 ± 188.40) compared to the ‘low’ condition (*M* = 494.83 ± 127.62, *p* = 0.002). Additionally, mean RTs in the ‘high’ condition (*M* = 682.64 ± 239.27) were significantly higher than in the ‘low’ condition (*M* = 494.83 ± 127.62, *p* < 0.001). For T4, the Friedman test indicated a significant effect of task demand on mean RT [*χ*^2^ (2) = 7.529; *p* = 0.023]. Mean RTs were significantly higher in the ‘high’ condition (*M* = 853.77 ± 206.57) compared to the ‘low’ condition (*M* = 702.99 ± 155.41, *p* = 0.030). Overall, these results demonstrate that increased task demand systematically leads to longer reaction times at the DRT in competition with each of the four tasks, with the most demanding conditions producing the most significant increases in mean RT.

##### 3.1.3.4 Effect of task demand on NASA-RTLX scores

**Figure 5:**
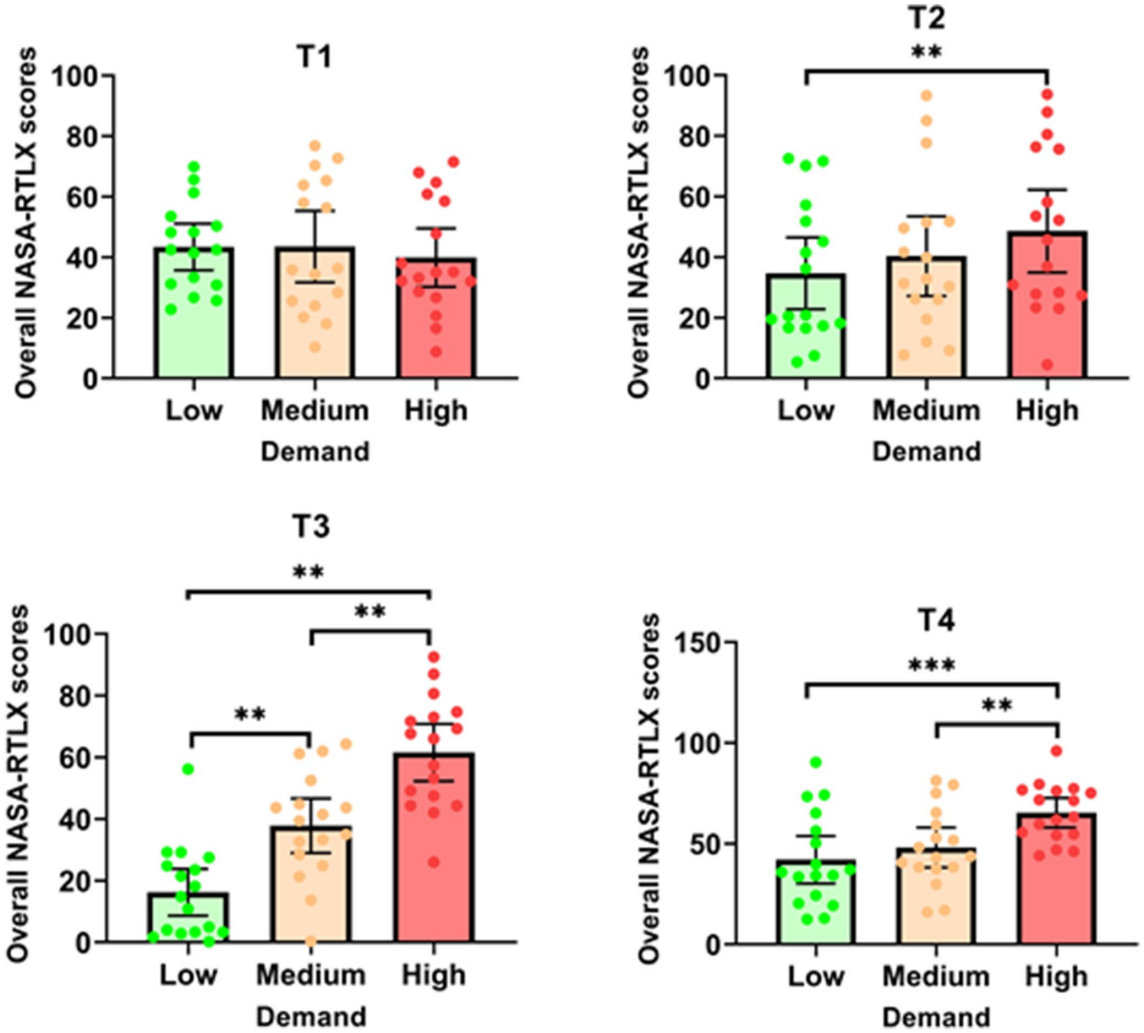
Overall NASA-RTLX scores for each task as a function of demand. *p < 0.05; **p < 0.01; ***p < 0.001.

The analysis of overall NASA-RTLX scores, which measure perceived workload, revealed varying effects of task demand across the different tasks (see Figure 5). For T1, there was no significant effect of task demand on overall NASA-RTLX scores [*F* (2, 30) = 0.629; *p* = 0.540]. In contrast, T2 showed a significant effect of demand on overall NASA-RTLX scores [*χ*^2^ (2) = 9.343; *p* = 0.009]. The scores were significantly higher in the ‘high’ demand condition (*M* = 48.55 ± 26.47) compared to the ‘low’ demand condition (*M* = 34.61 ± 23.00, *p* = 0.005). For T3, the Friedman test indicated a significant effect of task demand on overall scores [*χ*^2^ (2) = 32.118; *p* < 0.001], with significant differences between all task demand conditions (*p* = 0.015). Similarly, T4 demonstrated a significant effect of task demand on overall NASA-RTLX scores [*F* (2, 32) = 12.523; *p* < 0.001]. Scores were significantly higher in the ‘high’ demand condition (*M* = 65.30 ± 14.24) compared to both the ‘low’ demand condition (*M* = 41.99 ± 22.95, *p* < 0.001) and the ‘medium’ demand condition (*M* = 48.06 ± 19.34, *p* = 0.003). In summary, these findings indicate that increased task demand significantly elevates perceived workload for Tasks 2, 3, and 4, with T4 showing the most pronounced effect. However, T1 did not exhibit a significant change in perceived workload across different demand levels.

#### 3.1.4 Convergent validity results

Means and standard deviations of mean success rates for the four tasks, and the total, are presented in Table 3. Table 4 shows the correlation matrix between the mean success rate for each task, the total, and marks obtained in the TC1 and MC1 military exams. The convergent validity analysis of the multitask system found that none of the correlations between performance on the four tasks, and marks in the MC1 and TC1 exams were significant (see Table 4).

**Table 3:** Mean, standard deviation, minimum and maximum scores for each task and marks in the MC1 and TC1 exams.

Conditions	*M*	SD	Minimum	Maximum
Average T1	0.78	0.08	0.61	0.92
Average T2	0.83	0.09	0.70	0.97
Average T3	0.87	0.05	0.78	0.96
Average T4	0.72	0.1	0.55	0.87
Average of the 12 conditions	0.80	0.05	0.72	0.89
MC1	13.11	1.22	10.93	15.19
TC1	14.47	1.89	11.00	17.05

**Table 4:** Correlation matrix for the mean pass rate for each task, the total, and marks obtained in the TC1 and MC1 military exams.

Measures	MC1		TC1	
	Pearson’s *r*	*p*-value	Pearson’s *r*	*p*-value
Average T1	0.48	0.16	−0.09	0.82
Average T2	0.46	0.19	0.31	0.41
Average T3	−0.18	0.63	0.37	0.33
Average T4	0.08	0.83	−0.16	0.69
Average of the 12 conditions	0.46	0.18	0.18	0.65

*p < 0.05; **p < 0.01; ***p < 0.001.

#### 3.1.5 Content validity results for the simulated multitask environment

The analyses examined mean response scores for each question included in the content validity questionnaire (see Table 5). With regard to content validity, the scale for interpreting the intra-class correlation coefficient to assess inter-rater reliability was as follows: <0.50, poor; between 0.50 and 0.75, moderate; between 0.75 and 0.90 good; and above 0.90, excellent (Koo and Li, 2016). The intra-class correlation coefficient was 0.224 with a 95% confidence interval of 0.124 to 0.398, corresponding to a ‘low’ level of inter-rater reliability. This result is not surprising, as extreme and opposite values (i.e., 0/50 and 50/50) were observed for the same item, with almost all of them concerned. This calls into question the reliability of our questionnaire in capturing the participants’ point of view in terms of content validity. To make the analysis of the results more robust, the observed scores were classified according to the following taxonomy:

- Between 0 and 12.5: ‘strongly disagree’
- Between 12.5 and 25: ‘somewhat disagree’
- Between 25 and 37.5: ‘somewhat agree’
- Between 37.5 and 50: ‘strongly agree’.

**Table 5:** Mean, standard deviation, and minimum and maximum scores for each item of the content validity questionnaire.

Content validity questionnaire items	Mean	SD	Minimum	Maximum
Q1	20.88	13.81	0	50
Q2	22.18	15.53	0	47
Q3	23.35	15.55	0	46
Q4	26.18	16.89	0	50
Q5	22	14.77	0	50
Q6	23.19	12.67	0	45
Q7	28.19	12.94	4	50
Q8	31.82	12.74	2	50
Q9	32.41	14.88	0	50
Q10	17	13.67	0	40
Q11	27.06	15.94	0	50
Q12	24.23	16.69	1	50
Q13	33.06	14.81	0	50
Q14	21	14.46	2	45

Here, the aim was to estimate what percentage of participants indicated that they somewhat or strongly agreed with each proposition.

**Figure 6:**
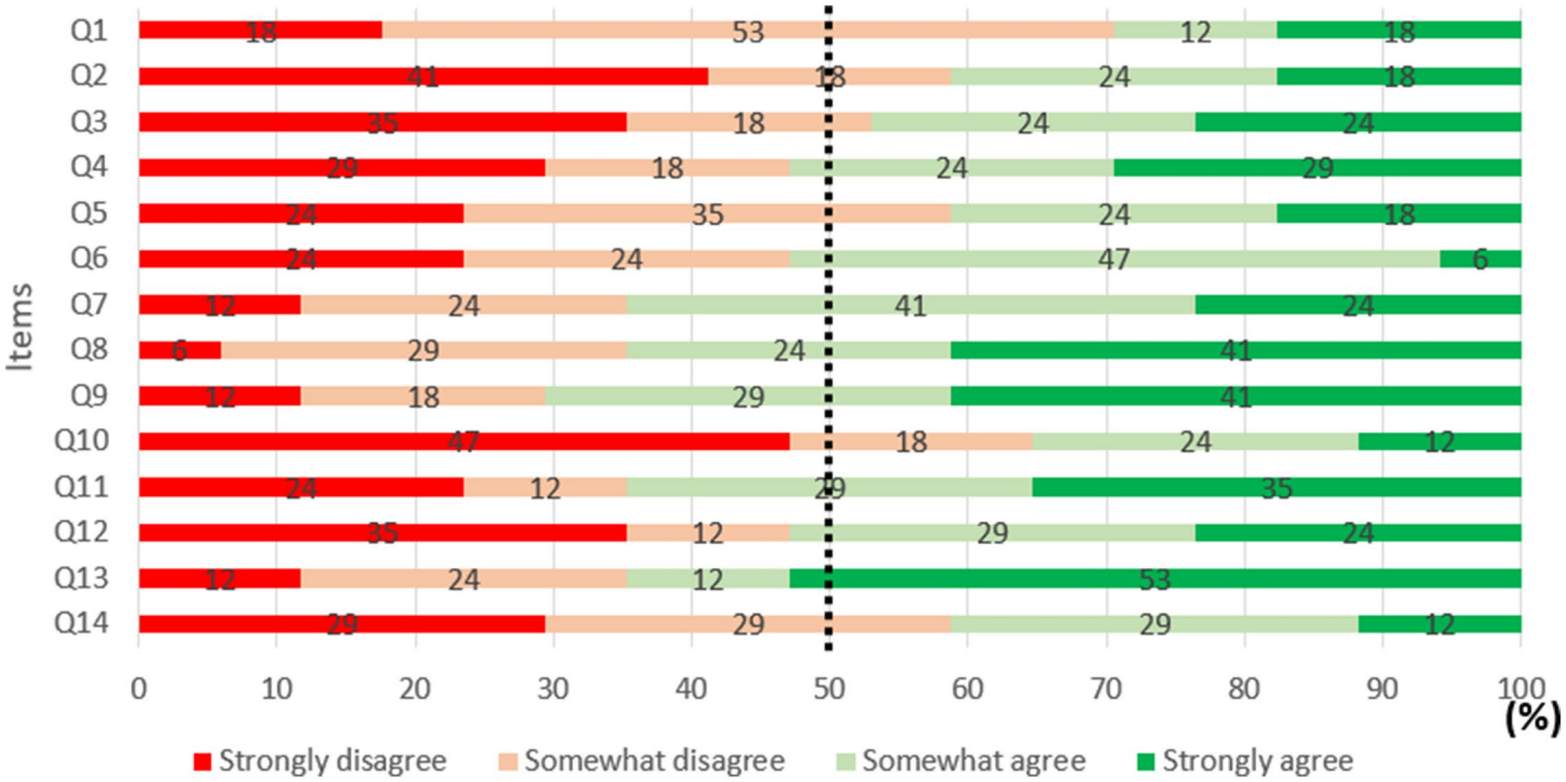
Percentage of agreement for each item included in the content validity questionnaire.

The results showed that for questions 4, 6, 7, 8, 9, 11, 12 and 13, at least 50% of responses were over 25/50 (see Figure 6). On the other hand, for questions 1, 2, 3, 5, 10 and 14, less than 50% of responses were above 25/50.

#### 3.1.6 Discussion

We designed and validated a microworld, called SMES, for assessing the cognitive load of infantry personnel in a multitasking situation. The design process was based on an activity analysis, and led to the identification of four generic tasks that were reproduced in the simulated task environment. Our experiment enabled us to assess the sensitivity of cognitive load measures, together with the simulator’s convergent and content validity.

##### 3.1.6.1 Sensitivity of measurements to variation in demand

The results show that performance on three tasks (T2, T3 and T4) deteriorated as demand increased. In these cases, the parameters chosen to vary task demand were, therefore, relevant. For T2, the mean angular error increased as the time to answer decreased. For T3, recall performance decreased as the amount of verbal information to be retained increased. This may be explained by high, or even excessive demand on the limited capacities of working memory, and, more specifically, the phonological loop, one of the components that holds verbal information in memory (Baddeley, 2003). For T4, group management deteriorated as the number of military targets to be protected, and the number of enemies increased. In contrast, for T1, participants remained equally effective even when the number of events to be detected increased (20, 40 or 80 items). In this case, the participant’s attentional resources were sufficient to meet the increased demand (Kahneman, 1973; Young et al., 2015), while this was not the case for the other tasks, where a deterioration in performance was observed. Thus, as task demand increases, task performance deteriorates. Hypothesis H1a is therefore supported for tasks T2, T3 and T4. It is not supported for task T1. For tasks T2, T3 and T4, objective and subjective levels of cognitive load increased with task demand, resulting, respectively, in longer RTs on the DRT, and higher NASA-RTLX scores. In the case of T1, although RTs for the DRT were also longer, NASA-RTLX scores were unaffected, as was task performance. This suggests that the participant was able to invest additional resources to maintain his or her level of performance on the primary, T1, task. Hypothesis H1b is thus supported, as the mean DRT reaction time increased. Hypothesis H1c is supported for T2, T3 and T4 (overall NASA-RTLX scores increased), but not for T1. Tactile DRT performance was shown to be sensitive to various sources of task demand, such as perceptual load for T1, temporal pressure for T2, memory load for T3 and complexity for T4. While these results support the hypothesis of attentional resources mobilized by these varied sources of demand (Oberauer, 2019), DRT also proves to be an effective tool for probing the spare capacity of the different resources mobilized by tasks of varied perceptual modalities (i.e., visuo-spatial and auditory-verbal), assumed to be independent (Wickens, 2002, 2008). Taken together, these results bring us closer to the idea of a “universal” measure of cognitive load (Abich, 2013).

##### 3.1.6.2 Convergent validity

The lack of a relationship between performance in the multitasking system and marks for the squad leader exams indicates that the assessment of the SMES’s convergent validity is inconclusive. However, this lack of correlation could be explained by the content of the exams (MC1 and TC1). The latter do not directly require the use of the cognitive functions underlying operational tasks, but rather theoretical or procedural knowledge, which the system does not call upon. Further investigations are needed to evaluate convergent validity more effectively. One option could be to estimate the correlation between observed performance with the SMES, and observed performance during military exercises that implement each task individually, based on more precise and better-defined criteria. This was not feasible in the current study. Hypothesis H2 is therefore not supported. On the other hand, it is possible that a multitasking situation that implements concurrent tasks, which is closer to what the squad leader does in real life, might be more predictive than examining each SMES task in isolation.

##### 3.1.6.3 Content validity

With regard to content validity, as the intra-class correlation coefficient was low, means were not very informative. This disagreement between participants may be due to differences in comprehension of the items, or to too much focus being placed on the realism of the environment. It therefore seems more relevant to assess, for each item, what percentage of participants indicated that they ‘somewhat’ (> 25/50) or ‘strongly’ (> 37.5/50) agreed. The majority of item scores were above 25/50, indicating fairly good content validity. Hypothesis H3 is therefore supported but we need to be aware that the participants were not all of the same opinion. For the dimension ‘mental representation of the situation’, low scores are not surprising (Q1: 30% of participants indicated that they ‘somewhat’ or ‘strongly’ agreed, Q2: 40%). In practice, these tasks do not require any military expertise, and were only designed to reproduce the cognitive processes underlying real-life tasks. For the dimension ‘attentional costs of tasks’, participants indicated that demands for T1 (Q3: 48%) and T3 (Q5: 42%) were not sufficiently representative of real-life situations. It is likely that the range of difficulty is the cause of this degree of disagreement. The most difficult conditions in T1 and T3 involved identifying and reporting a change at least every 3 sec (for T1), and holding three pairs of phonetic letters in working memory (for T3). These demands on attentional and memetic resources are far beyond what infantry personnel typically encounter in operations. It is possible that agreement would improve if the conditions proposed to participants were less demanding.

On the other hand, attentional costs for T2 (Q4: 53%) and T4 (Q6: 53%) were judged to be realistic. For the dimension ‘cognitive processes mobilized’, participants felt that all of the tasks accurately reproduced the cognitive processes involved in real-life tasks (Q7: 65%). Specifically, T1 (Q8: 65%), T2 (Q9: 70%) and T4 (Q11: 64%) were deemed representative of their real-life equivalents. On the other hand, participants felt that T3 did not require quite the same cognitive processes (Q10: 36%). The inability to take notes when using the SMES (when this is possible in reality) is probably a leading cause of disagreement, since note-taking draws upon other cognitive functions that are not represented in the SMES. With regard to the dimension ‘usefulness and practical scope’, participants indicated that the system would be useful to evaluate a squad leader’s skill level (Q12: 53%), and that it would make a good training tool (Q13: 65%). On the other hand, participants did not think that the system would help them learn to develop better ‘situational intelligence’ (Q14: 41%). This score is consistent with scores for the first dimension, as the SMES simulates a generic situation, while situational awareness is, by nature, specific to a particular situation and context. It involves attention, perception, and decision-making processes that enable the individual to construct a mental representation of the current situation (Endsley, 1995). Moreover, this construction is a function of the soldier’s expertise, which is not tested in the tasks that are included in the SMES.

##### 3.1.6.4 Summary of the results of experiment 1

Our results show that the cognitive load measures used are sufficiently sensitive. Hypotheses H1a, H1b and H1c are, in general, supported. The exception is T1, which was not sufficiently demanding even in the most demanding condition. The manipulated parameters are therefore relevant for varying demand in T2, T3 and T4. They will have to be adjusted for T1. As for convergent validity, Hypothesis H2 is not supported, as the results show that it is insufficient. On the other hand, Hypothesis H3 is supported, as the results demonstrate a satisfactory level of content validity.

Finally, it should be noted that the four tasks are the outcome of an approach that considers the multitasking situation as a set of interdependent tasks. However, exactly how the cognitive processes that are required when completing these tasks interfere with each other is a key characteristic of the multitasking situations encountered by the squad leader. This may explain the low level of convergent validity in Experiment 1. During Experiment 1, a key limitation was that it was necessary to assess the relevance of the manipulated demand parameters, and the validity of each task, separately. We therefore suggest that these tasks should be performed simultaneously, and that a multitasking performance score should be calculated in order to check whether this improves the SMES’s convergent validity (Vine et al., 2021).

### 3.2 Experiment 2: validation of the SMES in the multitasking setting

#### 3.2.1 Objective

In Experiment 2, participants were asked to complete the same tasks as in Experiment 1, but these tasks were presented simultaneously in order to generate multitasking situations. The aim was to verify that the SMES remained valid in multitasking situations.

We therefore: (1) evaluated the convergent validity of the SMES in a multitasking situation; (2) evaluated its content validity; and (3) compared responses to the content validity questionnaire in Experiment 1 with those in Experiment 2, in order to verify that the simultaneous task presentation improved the perceived validity of the SMES.

#### 3.2.2 Method

##### 3.2.2.1 Participants

Forty-one infantry squad leaders (38 men, 3 women) took part in the study. The mean age was 29.2 years (SD = 4.2); the mean time spent in the military was 8.79 years (SD = 4.67), and time as a squad leader was 3.09 years (SD = 2.41). All participants had normal vision and hearing, and volunteered to participate in the study. None of the participants in Experiment 2 had taken part in Experiment 1. The studies involving humans were approved by the Institutional review board OUEST V of Rennes and affiliated with the Center Hospitalier Universitaire (University Hospital Center) Pontchaillou (reference: CPP 2021-A00733-38 - CNIL 2021256 v 0). The studies were conducted in accordance with the local legislation and institutional requirements. The participants provided their written informed consent to participate in this study.

##### 3.2.2.2 Materials

###### 3.2.2.2.1 The SMES simulated task environment

The system was the same as the one used in Experiment 1, and included the same four tasks. Each condition consisted of a maximum of two or three concurrent tasks, in order to prevent cognitive overload. There was a risk that participants might switch from concurrent to sequential multitasking if demand became excessive, as the remaining cognitive resources only allowed them to perform one task at a time (Wickens, 2008; Wickens et al., 2015; Salvucci and Taatgen, 2011). We therefore chose not to present T2 and T3 simultaneously. In addition, a single level of demand was used for all multitasking situations, and for each task. These levels were chosen to equalize task difficulty as far as possible within the multitasking situations. If the difference in demand levels in competing tasks is too high, participants tend to prioritize the easier ones (Wickens et al., 2015, 2016). To avoid this bias, we selected demand levels for which DRT mean RTs were as similar as possible for the four tasks:

- T1: high demand (80 changes to be identified),
- T2: low demand (11 s to respond to each test),
- T3: medium demand (2-back),
- T4: low demand (2 military bases to protect, and only 25% of trials with two enemies to neutralize).

###### 3.2.2.2.2 Content validity questionnaire

The questionnaire used in Experiment 1 was used again (see description in section 3.1.2.2).

###### 3.2.2.2.3 Criteria adopted to evaluate convergent validity

As in Experiment 1, marks obtained in the MC1 and TC1 military exams were used as criteria to assess convergent validity.

##### 3.2.2.3 Experimental design and procedure

We adopted a repeated measures, two-factor design. Twelve, multitask situations lasting 5 min were presented to participants, to ensure that the duration of the two experiments was the same. Several task combinations were presented, depending on the number (2 or 3 simultaneous tasks), and perceptual modality (visuo-spatial and/or auditory-verbal) of the tasks. The SMES thus took the form of a test consisting of 12 conditions presented in random order to participants. The four concurrent task combinations were as follows:

- T1 + T2 + T4
- T1 + T3 + T4
- T3 + T4
- T2 + T4

This variability in multitasking situations was necessary to ensure the reliability of the construct measure (i.e., the ability to multitask in the infantry context). It also helped to limit the impact of specific or random factors linked to each situation, learning effects, and the same task prioritization strategy for all conditions. Using the same response strategy for all conditions could mask participants’ true level of ability. This diversity also helps to avoid redundancy in multitasking situations, which could degrade both motivation and vigilance. After completing a biographical questionnaire, participants were given instructions for each task. This was followed by familiarization with each of the four tasks. Once ready, the 12 experimental conditions were run in random order. Each condition lasted 5 min. When all had been run, participants completed the content validity questionnaire.

##### 3.2.2.4 Statistical analysis

Bravais–Pearson correlations between task performance and exam marks were used to assess convergent validity. An intra-class correlation test was used to estimate inter-rater reliability regarding responses to the content validity questionnaire. As in Experiment 1, the aim was to check whether means were interpretable, or whether it was preferable to interpret the mean agreement rate. Finally, with regard to the content validity questionnaire, a Chi-square test of homogeneity was used to check whether the percentages of participants in the ‘somewhat agree’ and ‘strongly agree’ categories for each item were identical for the two groups of participants, in other words, in single-task situations (Experiment 1) and in multitask situations (Experiment 2). The significance threshold was set at <0.05.

##### 3.2.2.5 Operational hypotheses regarding convergent and content validity

Convergent validity was evaluated by measuring the correlation between performance in the simulated multitasking environment, and marks for the MC1 and TC1 exams. The simulator’s content validity was evaluated using the same questionnaire used in Experiment 1. Our hypotheses were as follows:

- H3a: There should be a correlation between the performance obtained with the SMES and MC1 and TC1 exam marks (convergent validity);
- H3b: If content validity is acceptable, questionnaire scores should be above 25 (out of 50) for at least 50% of participants, for each item.

#### 3.2.3 Results

##### 3.2.3.1 Convergent validity in multitasking

We based our analyses on mean performance of the summed blocks, in other words, the experimental conditions (see Table 6). The variable denoted as 1–2 represents mean performance for the first two test blocks, 1–6 represents mean performance for blocks 1 to 6, and 1–12 represents mean performance for all test blocks. The results show that the correlation between the mean of the test blocks in the simulated task environment and the tested variables becomes significant from the mean of blocks 1–6, and stable from the mean of blocks 1–8 (see Table 7), with *r* coefficients corresponding to ‘medium’ and ‘large’ effects according to Cohen’s taxonomy (Cohen, 1988).

**Table 6:** Means and standard deviations of summed block performance in ascending order and marks in the MC1 and TC1 exams.

Summed blocks and marks	Mean	SD	Minimum	Maximum
1–2	0.56	0.12	0.28	0.81
1–3	0.59	0.11	0.3	0.84
1–4	0.59	0.11	0.38	0.82
1–5	0.6	0.1	0.38	0.8
1–6	0.6	0.09	0.4	0.78
1–7	0.61	0.09	0.41	0.76
1–8	0.62	0.08	0.41	0.78
1–9	0.63	0.09	0.4	0.8
1–10	0.63	0.09	0.4	0.79
1–11	0.64	0.09	0.39	0.78
1–12	0.64	0.09	0.4	0.78
MC1	14.94	1.24	12.86	17.06
TC1	15.32	1.18	14	18.41

**Table 7:** Correlation matrix between mean summed block performance and MC1 and TC1 marks.

Measures	MC1		TC1	
	Pearson’s *r*	*p*-value	Pearson’s *r*	*p*-value
1–2	−0.1	0.67	0.36	0.14
1–3	0.05	0.83	0.44	0.07
1–4	0.09	0.72	0.46	0.06
1–5	0.2	0.41	0.42	0.09
1–6	0.32	0.17	0.5	0.036*
1–7	0.38	0.1	0.45	0.06
1–8	0.38	0.1	0.51	0.029*
1–9	0.4	0.08	0.51	0.031*
1–10	0.42	0.07	0.49	0.039*
1–11	0.49	0.029*	0.5	0.036*
1–12	0.51	0.022*	0.51	0.031*

*p < 0.05; **p < 0.01; ***p < 0.001.

##### 3.2.3.2 Content validity in multitasking

**Figure 7:**
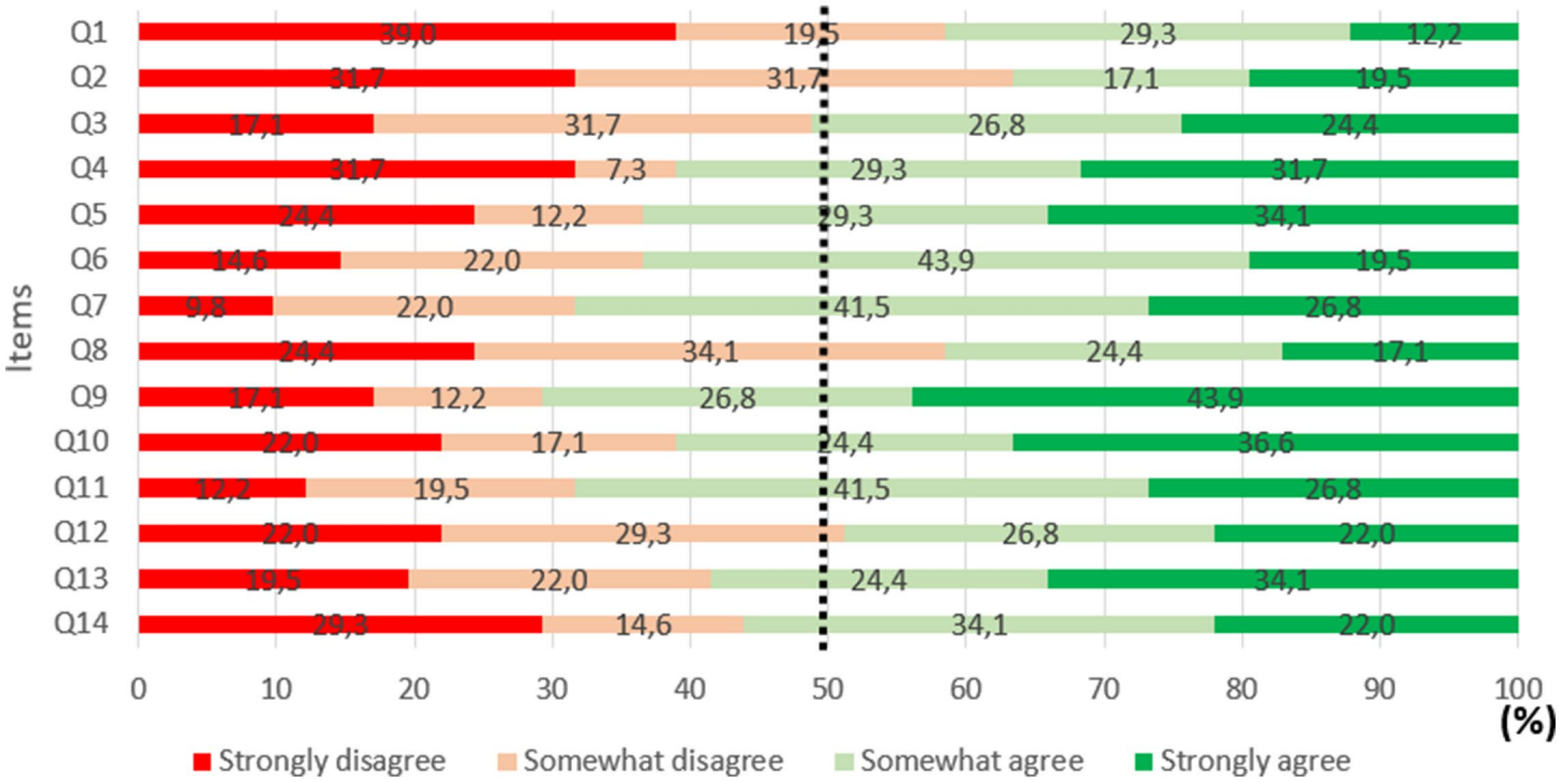
Percentage of agreement for each item included in the content validity questionnaire.

The analyses examined mean response scores for each question included in the content validity questionnaire (see Table 8). The scale used to interpret the intra-class correlation coefficient was the same as that used in Experiment 1 (Koo and Li, 2016). The intra-class correlation coefficient was 0.393, with a 95% confidence interval of 0.303 to 0.506, corresponding to a ‘poor’ level of inter-rater reliability. As reliability was low, observed scores were classified according to the taxonomy used in Experiment 1, namely the aim was to estimate what percentage of participants indicated that they ‘somewhat’ or ‘strongly’ agreed with each proposition. The results show that for items 3–7, 9–11, 13 and 14, at least 50% of responses were above 25/50 (see Figure 7). On the other hand, for questions 1, 2, 8 and 12, less than 50% of responses were above 25/50. The result shows no significant difference between the two distributions [*χ*^2^ (13) = 4.964, *p* = 0.976].

**Table 8:** Means and standard deviations of scores in response to the content validity questionnaire.

Content validity questionnaire items	Mean	SD	Minimum	Maximum
Q1	20.07	14.77	0	48
Q2	21.93	14.97	0	50
Q3	25.78	13.18	0	50
Q4	26.83	18.13	0	50
Q5	27.83	15.25	0	50
Q6	27.51	14.16	0	50
Q7	29.71	12.36	0	50
Q8	23.44	14.46	0	50
Q9	30.85	15.61	0	50
Q10	28.51	15.74	0	50
Q11	28.93	13.36	0	50
Q12	24.63	15.05	0	47
Q13	27.51	16.18	0	50
Q14	24.98	16.15	0	50

#### 3.2.4 Discussion

As the sensitivity of the measures to variation in demand had been verified in Experiment 1, here, we only test convergent and content validity in multitasking situations. Results for content validity were also compared between Experiments 1 and 2.

##### 3.2.4.1 Convergent validity in multitasking

Experiment 2 demonstrated that performance in the SMES tasks predicted TC1 and MC1 marks. The relationship between mean performance in multitasking sessions, on the one hand, and TC1 and MC1 marks, on the other, became significant and stable from the eighth block of tests for TC1, and the eleventh block of tests for MC1.

With regard to the lack of a relationship between TC1 marks and performance on test blocks 1–7, and between MC1 marks and performance on test blocks 1–10, we believe that the phase of familiarization with the SMES interface may have played a role. The time allocated to each task enabled participants to understand what was required, and perform well from the start of the experiment, but it is likely that some time was required to reach this level of familiarity. How the participant interacts with the SMES is unlike how infantry personnel interact with a real environment. Although the familiarization period clearly increases measurement error, it applies equally to all participants, and can be disregarded.

##### 3.2.4.2 Content validity in multitasking

As in Experiment 1, given that the intra-class correlation coefficient was low, we assessed the percentage of participants who indicated that they ‘somewhat’ or ‘strongly’ agreed with each item. The majority of scores were above 25/50, once again indicating good content validity.

For the dimension ‘mental representation of the situation’, low scores are not surprising (Q1: 41.5% of participants indicated that they ‘somewhat’ or ‘strongly’ agreed, Q2: 36.6%). In practice, these tasks do not require any military expertise, and were only designed to reproduce the cognitive processes underlying real-life tasks. For the dimension ‘attentional costs of tasks’, participants indicated that demands in the four tasks T1 (Q3: 51.2%), T2 (Q4: 61%), T3 (Q5: 63.4%) and T4 (Q6: 63.4%) were sufficiently representative of reality. For the dimension ‘cognitive processes mobilized’, participants felt that T1 (Q8: 41.5%) was not representative of the real-life task. This can be explained by the fact that only the most difficult level was presented (i.e., detect an event occurring every 3 sec). This level of difficulty was perceived as excessive by participants. Regarding the other three tasks, participants felt that they sufficiently reproduced all of the cognitive processes involved in real-life tasks (Q7: 68.3%), and they were deemed representative of their real-life equivalents (T2 - Q9: 70.7%; T3 - Q10: 61%; T4 - Q11: 68.3%). For the dimension ‘usefulness and practical scope’, participants indicated that the system would not be useful to evaluate a squad leader’s level of competence (Q12: 48.8%), but that it would make a good training tool (Q13: 58.5%), and would help people learn to develop better ‘situational intelligence’ (Q14: 56.1%).

##### 3.2.4.3 Conclusion of experiment 2

Hypotheses H3a and H3b are supported, with convergent and content validity, respectively, demonstrated in multitasking situations. With regard to content validity, overall, results for Experiment 2 were similar to those of Experiment 1. Carrying out the tasks individually was therefore enough to ensure content validity, and this was not improved in a multitasking situation.

## 4 General discussion

A design approach, based on an analysis of real-life activity, led to the development of a simulated multitasking environment, the SMES. The tasks that were designed were intended to reproduce the cognitive processes used in the operational multitasking situations encountered by infantry personnel.

### 4.1 Regarding SMES validity in multitasking situations

Convergent validity, in particular, improved in multitasking situations. The multitasking situations presented in Experiment 2 were able to reproduce the interference between cognitive processes found in real life. This could explain why, in this case, performance predicts the exam results obtained by participants. Furthermore, although it would appear that it is the ability to multitask, rather than each of the tasks taken individually that is predictive of the chosen criteria (Barron and Rose, 2017), it remains necessary to test whether it is a general multitasking ability, or an ability related specifically to the SMES tasks that predicts the selected criteria. In practice, multitasking ability could be explained by working memory capacity, fluid intelligence or attention control (Redick et al., 2016). One reason for the lack of convergent validity in Experiment 1 could be that the cognitive load in the monotasking situation was insufficient for performance scores to be sensitive to the individual’s level of ability, and consequently predictive of the chosen criteria. In practice, multitasking situations generate higher cognitive load than monotasking situations, particularly when the tasks share the same perceptual modalities or use the same type of codes (Wickens, 2008).

The assessment of the content validity of the SMES showed that while the task characteristics were reliable reproductions of their real-life equivalent, whether in a single-task or multitask situation, there was room for improvement. In particular, T1 was judged to be insufficiently representative in terms of attentional cost in a single-task situation, and in terms of cognitive processes in a multitask situation. This is clearly due to the fact that the demands of T1 are higher in the SMES than in reality, particularly in the multitasking situation where the most difficult condition was selected. However, this change detection task is very important for infantry personnel, as they need to be able to identify potential threats as they arise during a mission. Even if, in reality, there are fewer changes, the SMES appears to be a suitable training tool. With regard to T3, we felt that it was insufficiently representative in terms of attentional cost and cognitive processes in a single-task situation. The n-back task may, in fact, be more difficult than the situations that are encountered in real life (notably, 3-back), especially as the SMES made it impossible for participants to take notes, as they do in real life. In the multitasking situation, the choice of the moderately demanding condition (2-back) is likely to have canceled out this effect, as T3 was deemed representative in terms of attentional cost and cognitive processes. It is also important to note that while the tasks included in the SMES reproduce the cognitive processes of squad leaders, they do not call on their military expertise.

With regard to the use of SMES as a training tool, caution is called for. Studies frequently show that cognitive training, and working memory training in particular, is not very effective in transferring benefits to other tasks or situations (Draheim et al., 2022). On the other hand, attentional control has shown encouraging results for this transfer of benefits to real-life situations (Draheim et al., 2022) and has been correlated with general multitasking ability (Redick et al., 2016). It is therefore possible that a training program including SMES to improve real-life performance could be effective (i.e., enable far transfer), if SMES effectively mobilizes general multitasking ability. To extend the SMES validation process, it would be interesting to compare its predictive capabilities with other simulated multitasking environments, such as MATB-II or SynWin (Elsmore, 1994; Hambrick et al., 2010), and verify whether correlations are higher with the SMES. This would confirm how effective the design approach described above is able to simulate the characteristics of real-life tasks undertaken by squad leaders.

### 4.2 Limits

Certain limitations should be noted regarding the criteria used in the evaluation of convergent validity, namely marks obtained on the TC1 and MC1 infantry squad leader exams. Firstly, these exams do not focus exclusively on the four main tasks reproduced in the SMES, and they include dimensions that are not tested by it. However, it was not possible to create specific marked exercises in the training field for the purposes of our study. Secondly, it is possible that a sampling bias was present, as only participants with marks above 10 out of 20 were able to become a squad leader, and, thus, participate in our study. Moreover, some participants did not have access to this information, and were unable to send us their exam marks, which may also have contributed to this bias. It is therefore important to bear in mind that the predictive power of SMES performance could be enhanced by optimizing the collection of exam results.

Another limitation is that randomizing experimental conditions across participants meant that they did not all experience the same multitask situations when not all conditions were included, as multitask situations varied with respect to the number of tasks and their perceptual modality. Although this may introduce measurement error, and reduce the reliability of content validity indicators and the learning effect, it is not a threat to the internal validity of our study. On the contrary, it may explain why the tool’s convergent validity increases as the number of trials increases. It should be noted that this variability falls as the number of runs increases, up to the twelfth and last run, when all participants have been exposed to all experimental conditions.

Also, the low intra-class correlation coefficient is indicative of a lack of robustness in the content validity questionnaire used. It is possible that there were inconsistencies in the understanding of the questions, features of the tool really evaluated by the raters (i.e., realism rather than similarity of the cognitive processes involved in the tasks) as well as response biases (e.g., social desirability and extreme response bias) (Furnham, 1986), with operational populations being particularly vulnerable to these (Thunholm, 2001). These factors may explain the discrepancies in responses. This raises the interest of developing a subjective and standardized measure of the content validity of simulated task environments. To our knowledge, such a tool does not yet exist, as custom-made tools are created for each new simulated task environment designed (e.g., Classen et al., 2020; Medeiros et al., 2021; Varoquier et al., 2017).

With regard to the use of DRT, a distinction should be made between discrete and continuous tasks. Depending on the rate at which the task stimuli are presented, the temporal gap between two participant responses and the duration of the trials, the performance indicators are not sampled identically. For example, the sampling rate for the DRT response is between 3 and 5 s, while that for the PTSOT task (T2) is between 7 and 11 s. Thus, interference of task responses at the central and/or motor (i.e., manual) level may have occurred uncontrollably between conditions and contributed to the deterioration of task performance. As cognitive load is a dynamic phenomenon, loss of information is possible.

### 4.3 Future uses for the SMES

In this study, we focused on the effects of concurrent multitasking on cognitive load. However, SMES could well be used to explore research questions relating to sequential multitasking and task interruption, which also concern squad leaders (Chérif et al., 2018). For sequential multitasking, it is possible to manipulate the temporality of tasks so that the participant performs only one task at a time, alternating from one to the other. It is possible to interrupt a task in progress and present a new, unexpected task to study the effects of task interruption on cognitive load. Basically, SMES could be modified to study these three forms of multitasking situation. The use of SMES for selection can be envisaged, while interest in multitasking ability and its potential in professional selection processes is recent and growing (Hilla et al., 2024). Studies have shown that assessing multitasking abilities, either through dual-task paradigms or multitasking scenarios, can predict success in military operations (Hilla et al., 2024). The research highlighted the importance of multitasking assessments in selecting candidates for military roles involving multitasking, such as pilot positions, where multitasking performance predicts success in flight and academic exams (Barron and Rose, 2017).

In an extension to our SMES validation research, we plan to use the system to: (i) understand the effect of various factors (such as the level of demand and perceptual modalities) on the cognitive processes used by infantry personnel during operations; and (ii) extrapolate these results to real-life situations. The tasks included in the SMES could be adapted to answer other research questions. It is entirely possible to re-use our criteria to assess the convergent validity of the SMES with new tasks. It would also be interesting to repeat our experiments with interacting, or interdependent tasks (e.g., information from the communication and orientation tasks is needed to be able to manage the two teams in the tactical decision-making task). The implementation of two tasks that share a common goal would support the systematic use of cognitive processes. It would also be able to account for mutual influences, and thereby make the individual’s internal state more representative of real-life situations. Previous work has already demonstrated different results for dependent and interdependent tasks (Donmez et al., 2009; Smit et al., 2017). However, any modification of task characteristics that does not stem from an analysis of real-life tasks could alter the tool’s current validity. Indeed, it is necessary to determine under what circumstances the tool can be adapted to new research questions without altering its psychometric qualities. As SMES reproduces generic, non-specific tasks, its use has the potential to be easily extended to other populations, notably in the army (e.g., artillery). Future studies could test the generalizability of SMES by comparing different populations. In addition, it seems relevant to consider the contributions of the paradigms of situated and embodied cognition. The aim of such an approach would be to extrapolate the results observed in the laboratory to real-life situations.

The results of a simulation that includes measures of cognitive load could vary considerably if constraints that are present in real-life situations are taken into account. For example, infantry squad leaders are often required to carry heavy loads (e.g., a 30 kg rucksack), engage in intense physical activity, and work in extreme temperatures. These conditions consume a lot of energy, and lead to fatigue (Weeks et al., 2010). Carrying a load has also been found to affect performance on a vigilance task (Mahoney et al., 2007) and cognitive performance (Eddy et al., 2015). Taking these parameters into account in experimental protocols could improve the predictive ability of performance observed with the SMES. Psychometrics is also often used for selection purposes, particularly in the workplace. Multitasking skills are critical in the military environment (Chen and Terrence, 2008; Chérif et al., 2018). The SMES could be a useful addition to certain tests used in the professional evaluation of squad leaders. In addition, it would be interesting to evaluate the construct validity of the system by comparing the performance of civilian and military participants. However, as we have demonstrated, the validity of the system, and the sensitivity of the measures are sufficient for it to form the basis for future experiments. The SMES holds potential for advancing our understanding of the intricate dynamics influencing cognitive performance in high-stakes operational scenarios.

## Data Availability

The original contributions presented in the study are included in the article/Supplementary material, further inquiries can be directed to the corresponding author.
