# Supplementary material for: Design and validation of a simulated multitasking environment for assessing the cognitive load on the infantry squad leader
Source: Front Psychol. 2024 Aug 27;15:1433822. doi: 10.3389/fpsyg.2024.1433822 (PMC11385692; doi:10.3389/fpsyg.2024.1433822)
Supplement: Supplementary file 10 [file Table_10.DOCX]

Supplementary Material

# Content validity questionnaire

| Dimensions | No. | Items |
| --- | --- | --- |
| Mental representation of the situation | Q1 | I felt as if I encountered the same difficulties while performing the tasks in the simulator as I would in a field mission. |
|  | Q2 | I had the same situational awareness in the simulator as I would have in a field mission. |
| Attentional costs of tasks | Q3 | The mental workload during the map surveillance task was equivalent to what I typically feel when monitoring changes in the tactical situation during a field mission. |
|  | Q4 | The mental workload during the spatial orientation task was equivalent to what I typically feel when navigating using a map and compass during a field mission. |
|  | Q5 | The mental workload during the information transmission task was equivalent to what I typically feel when transmitting information via radio during a field mission. |
|  | Q6 | The mental workload during the tactical decision-making task was equivalent to what I typically feel when making tactical decisions during a field mission. |
| Cognitive processes involved | Q7 | While I’m aware that the simulator tasks are simplified, I thought that they closely mimic the cognitive processes involved in real tasks performed by a group leader in the field. |
|  | Q8 | For the map surveillance task: I felt that paying attention to changes on the map required the same kind of thinking as in real-life when I have to pay attention to changes in the tactical situation. |
|  | Q9 | For the spatial orientation task: I felt that estimating the direction of the symbol required the same kind of thinking as when I have to orient myself or estimate the direction of something in the field. |
|  | Q10 | For the message transmission task: I felt that memorizing and transmitting radio messages made me think in the same way as when I have to memorize and transmit information over the radio network during a field mission. |
|  | Q11 | For the tactical decision-making task: I felt that commanding my teams and devising a strategy to neutralize enemies required the same kind of thinking as when I have to develop a maneuver or tactic in a field mission. |
| Utility and practical significance | Q12 | I believe that this simulator is useful for assessing the level of competence of a group leader. |
|  | Q13 | I believe that this simulator is a valuable training tool for group leaders. |
|  | Q14 | I believe that training on this simulator would enhance my situational awareness in the field. |
